# Examination of the interior of sand fly (Diptera: Psychodidae) abdomen reveals novel cuticular structures involved in pheromone release: Discovering the manifold

**DOI:** 10.1371/journal.pntd.0009733

**Published:** 2021-12-21

**Authors:** Gabriel B. Tonelli, José D. Andrade-Filho, Aldenise M. Campos, Carina Margonari, Amanda R. Amaral, Petr Volf, Elisabeth J. Shaw, James G. C. Hamilton

**Affiliations:** 1 Grupo de Estudos em Leishmanioses, Instituto René Rachou, FIOCRUZ Minas, Brasil; 2 Department of Parasitology, Faculty of Science, Charles University, Prague, Czech Republic; 3 Division of Biomedical and Life Sciences, School of Health and Medicine, Lancaster University, Lancaster, United Kingdom; Instituto Oswaldo Cruz, BRAZIL

## Abstract

The males of many species of New World Phlebotomines produce volatile terpenoid chemicals, shown in *Lutzomyia longipalpis* s.l. to be sex/aggregation pheromones. Pheromone is produced by secretory cells which surround a cuticular reservoir which collects the pheromone and passes it through a cuticular duct to the surface of the insect. The pheromone then passes through specialised cuticular structures on the abdominal surface prior to evaporation. The shape and distribution of the specialised structures are highly diverse and differ according to species. In this study we used SEM to examine the interior cuticular pheromone collection and transport structures of 3 members of the *Lu*. *longipalpis* s.l. species complex and *Migonemyia migonei*. We found a new structure which we have called the manifold which appears to be a substantial extension of the interior tergal cuticle connected in-line with the cuticular duct and reservoir. The manifold of the Campo Grande member of the complex is longer and wider than the Jacobina member whereas the manifold of the Sobral member was shorter than both other members of the complex. Overall, the secretory apparatus of the Sobral member was smaller than the other two. The manifold of *M*. *migonei* was very different to those found in *Lu*. *longipalpis* s.l. and was positioned in a pit-like structure within the tergal cuticle. The secretory reservoir was connected by a short duct to the manifold. Differences in the size and shape of the manifold may be related to the chemical structure of the pheromone and may have taxonomic value. Examination of the interior cuticle by SEM may help to locate the secretory apparatus of vector species where pheromonal activity has been inferred from behavioural studies but the external secretory structures or pheromones have not yet been found.

## Author summary

The males of many species of New World sand flies produce volatile chemicals shown in *Lutzomyia longipalpis* s.l. to be attractive to females and other males. These sex/aggregation pheromones are produced by secretory cells which surround a cuticular reservoir that collects the pheromone and from which it passes through a cuticular duct and a specialised structure on the surface of the abdomen, from where it evaporates. We examined by SEM the structure of the interior cuticular secretory apparatus of 3 members of the *Lutzomyia longipalpis* s.l. species complex and *Migonemyia migonei* and found a new structure associated with pheromone release that we have called the manifold. The manifold appears to be a substantial extension of the interior tergal cuticle that is connected in-line with the duct and reservoir. Significant differences in size and shape of the manifold between members of the *Lu*. *longipalpis* complex were observed. The *M*. *migonei* secretory apparatus were substantially different in appearance to those of *Lu*. *longipalpis* with the manifold positioned in a pit-like structure within the tergal cuticle. These differences which may have taxonomic value and may be related to the chemical structure of the pheromone or phylogenetic differences.

## Introduction

Members of the sand fly species complex, *Lutzomyia longipalpis* s.l. (Lutz and Neiva, 1912) are vectors of the Protist parasite *Leishmania infantum* Nicolle, 1908, the etiological agent of visceral leishmaniasis (VL) [1–3]. There is a close relationship between the distribution of VL cases and the distribution of *Lu*. *longipalpis* s.l. throughout most of Brazil and it has been proposed that the urbanization of members of this complex and it’s anthropophilic behaviour have increased the incidence of VL in many Brazilian states [4,5].

In some regions of Brazil, where *Lu*. *longipalpis* is not the most abundant sand fly species, VL cases are associated with another incriminated vector, *Migonemyia migonei* (França, 1920) [6,7]. This species is considered to be a potential vector because of its’ distribution, prevalence, anthropophily and the detection of *Leishmania infantum* DNA in blood-fed females [6]. In addition, based on evidence of the development of late-stage parasite forms in artificially infected sand flies this species is considered permissive for transmission of *Le*. *infantum* [8]. *Migonemyia migonei* has also been implicated as a vector of *Le*. (*V*.) *braziliensis*, the etiological agent of cutaneous leishmaniasis in different Brazilian regions [9,10].

Male *Lu*. *longipalpis* s.l. produce sex/aggregation pheromones which when present with host odour are attractive to conspecific males [11–13] and lead to the formation of leks on or near host animals where the males compete with each other for access to mating opportunities [14,15]. Females are attracted by the combination of the male produced pheromone and host odour [16–18]. They arrive at the lekking site after the males [17], choose a mate [14] take a blood-meal and depart [19]. It has been proposed that synthetic sex/aggregation pheromone co-located with insecticide could be used for vector control [20–22]. Pheromones have been used as a taxonomic tool to differentiate between morphologically identical members of New Zealand Tortricinae moths [23]. The sex/aggregation pheromones of *Lu*. *longipalpis* can also be used to differentiate between members of the *Lu*. *longipalpis* species complex [16,24–26].

In *Lu*. *longipalpis* s.l. the sex/aggregation pheromone has been associated with cuticular structures on the surface of tergites III or III and IV and associated with underlying glandular tissue where they have the visual appearance of either 1 or 2 pale spots (1S or 2S) [16,27]. Under SEM the external cuticular structures appear as small round elevations (papules) (mean diameter 3.0–3.5 um) with a central pore (mean diameter 0.25 μm) and a density of 21±2 (1S) or 19±2 (2S) 1000 μm^-2^ [28,29]. The pheromone is believed to be produced by the glandular cells that underly the papules and to be passively transported to the surface pores via a cuticular duct [27,29]. Five members of the *Lu*. *longipalpis* complex can be distinguished based on qualitative and quantitative differences in their sex/aggregation pheromones [24]. Two members have been characterised as producing methylsesquiterpene (C16) sex/aggregation pheromones and two members produce diterpene (C20) sex/aggregation pheromones [16,30,31]. *Lutzomyia longipalpis* from Lapinha (MG) produces significantly less (*S*)-9-methylgermcrene-B, than those from Sobral (CE) and is thus identifiable as a fifth member of the complex [24]. The characterisation of the complex as having these 5 putative members is supported by identification of molecular correlates (SNPs and CNVs) in the chemosensory genome [32].

Possible pheromone associated tergal structures have also been observed in other sand fly species where they occur in a variety of forms [13,19]. For example, in *Evandromyia lenti* and *E*. *carmelinoi*, apple-shaped structures with a central pore are present on the V and VI tergal segments [29]. Terpenoids including oxygenated compounds are produced in some of these other species e.g. *E*. *lenti*, *Lu*. *lichyi*, *Lu*. *cruciata*, [33–35] but they do not appear to be present in all those species that have structures [35]. Behavioural evidence for pheromonal activity for any of these compounds is limited to *Lu*. *longipalpis* and *Lu*. *cruciata* [33,36]. SEM analysis of the tergal structures in *M*. *migonei* have revealed that they form a shallow crater (average diameter ca. 3.2 μm) with a central pit (av. diameter ca. 0.4 μm) containing a central spike (height ca. 0.2 μm) within it [13,19,37]. There is some behavioural evidence that this species also produces a sex aggregation pheromone [37].

The presence of internal cuticular structures i.e. the reservoir and cuticular duct associated with the end apparatus and secretory cells has been revealed by TEM studies [27,29,38], there has been no SEM investigation of the internal cuticular structures in sand flies. Therefore, this SEM study was undertaken to investigate the internal cuticular structures associated with pheromone production and release and to compare the morphology of these structures in three members of the *Lu*. *longipalpis* species complex and *M*. *migonei*.

## Material and methods

### Ethics statement

Sand fly blood feeding at Lancaster University for colony maintenance was performed according to the guidelines and regulations of the Animals in Science Regulation Unit (ASRU) and in accordance with the terms of a regulated licence (PPL P2DB5013A) and in compliance with the Animals (Scientific Procedures) Act (ASPA) 1986 (amended 2012) regulations and was consistent with UK Animal Welfare Act 2006. All procedures involving animals were reviewed and approved by the Faculty of Health and Medicine Ethical Review Committee (FHMREC15125) at Lancaster University. Sand fly blood feeding at Charles University for colony maintenance was performed in accordance with institutional guidelines and Czech legislation (Act No. 246/1992, amendment No. 359/2012) which complies with relevant European Union guidelines for experimental animals. All procedures involving animals were approved by the Committee on the Ethics of Laboratory Experiments of the Charles University (Registration Number: MSMT-8604/2019-6).

### Sand flies

The male *Lu*. *longipalpis* used in the study were obtained from colonies held at Lancaster University, UK and the *M*. *migonei* were obtained from a colony held at Charles University, Czech Republic. The *Lu*. *longipalpis* males were representative of 3 of the 5 members of the species complex [24,30,32] and were established from females originally collected using miniature CDC light traps in chicken shelters (Table 1). The *M*. *migonei* colony was established from material originally collected using CDC light traps in Baturité, Ceará State, Brazil (04°19′41″S, 38°53′05″W). The *Lu*. *longipalpis* colonies were maintained in an insectary (28±2°C, 80±5% RH and a 12:12 light:dark (L:D) photoperiod) and all males used in this study were 7d old and classified as two spot (2S) [39]. The *M*. *migonei* colony was maintained under slightly different conditions (25–26°C, 70–95% RH and a 14:10 L:D photoperiod) [40] and males used were 5-7d old.

**Table 1:** Original collection site and pheromone type of the members of the *Lu*. *longipalpis species* complex held at Lancaster University used in the study.

collection locality	grid reference	pheromone type
Campo Grande—MS	20° 28’S, 54° 37’W	(*S*)-9-methylgermacrene-B (9MGB)
Jacobina—BA	11° 11’S, 40° 31’W	3-methyl-α-himachalene (3MH)
Sobral—CE	3° 41’S, 40° 20’W	Sobralene (SOB)

The male sand flies used in this study were removed from the colony and killed by placing them in a freezer (-5°C) for 20 mins. They were then placed in a plastic screwcap vial and covered with a few drops of ethanol (70%) and stored (-20°C) until dissection.

### Dissection

To prepare the male sand fly abdomen for SEM, a male was placed in a drop of saline solution (1% w/v) on a glass microscope slide. The entire abdomen was removed from the thorax with entomological needles under a dissecting microscope (Stemi 508, Carl Zeiss Ltd, Cambridge, UK). The interior of the whole abdomen or the abdominal segments III and IV (excised from the other abdominal segments) were then exposed by a further dorsoventral incision.

### Digestion, cleaning and drying of cuticle sections

To remove the internal soft tissue covering the interior cuticular structures we submerged the dissected abdominal samples in 10% (w/v) KOH [41] in glass Petri dishes placed on a plate rocker. *Lutzomyia longipalpis* samples were digested in KOH for 4 hours and *M*. *migonei* were digested in KOH for 24 hours. After the KOH digestion, the samples were washed in saline solution (1% w/v) in a Petri dish for 5 mins (3 times) followed by a final rinse in distilled water. The samples were then dehydrated by washing in alcohol (50%, 70%, 90% and 100%) for 5 mins each and then left overnight in a fume hood in hexamethyldisilazane until completely dry.

### Scanning electron microscopy (SEM)

After the digestion, cleaning and drying, samples were mounted on SEM stubs with double sided adhesive tape and sputter coated with gold (20nm) (Edwards S150A; Edwards UK, Burgess Hill, UK). The samples were then examined with a scanning electron microscope (JEOL JSM-7800F and JEOL JSM-5600; Jeol (UK) Ltd, Welwyn Garden City, UK) operated at 18kV. In total nine Campo Grande, eight Sobral and seven Jacobina *Lu*. *longipalpis* specimens as well as nine *M*. *migonei* specimens were prepared and examined by SEM.

### Secretory apparatus measurements

We measured; manifold width, manifold length, reservoir and cuticular duct length, and secretory apparatus length in 10 randomly selected secretory apparatus from five individuals of each of the three *Lu*. *longipalpis* pheromone types (total measurements = 600) using Image J software.

Comparison of the size of the different components of the secretory apparatus measured in each of the three *Lu*. *longipalpis* chemotypes and *M*. *migonei* was made by Generalized Linear Model (GLM). We assumed that there was no difference in the morphology of the structures of individuals originating from the same location. The dimensions of each part of the structure measured were used as response variables, while the colonies were considered to be explanatory variables. Tukey’s test was used to determine which measurements were different from each other. All the models were made using R (v3.6.1, R Development Core Team 2016), followed by residual analysis to standardize the data distribution.

## Results

### The *Lutzomyia longipalpis* complex secretory apparatus

Preliminary investigation showed that digestion of sand fly samples in KOH (10%) over 4 hours removed tissue from the inner cuticular surface of the abdomen without damaging the target structures.

**Fig 1:**
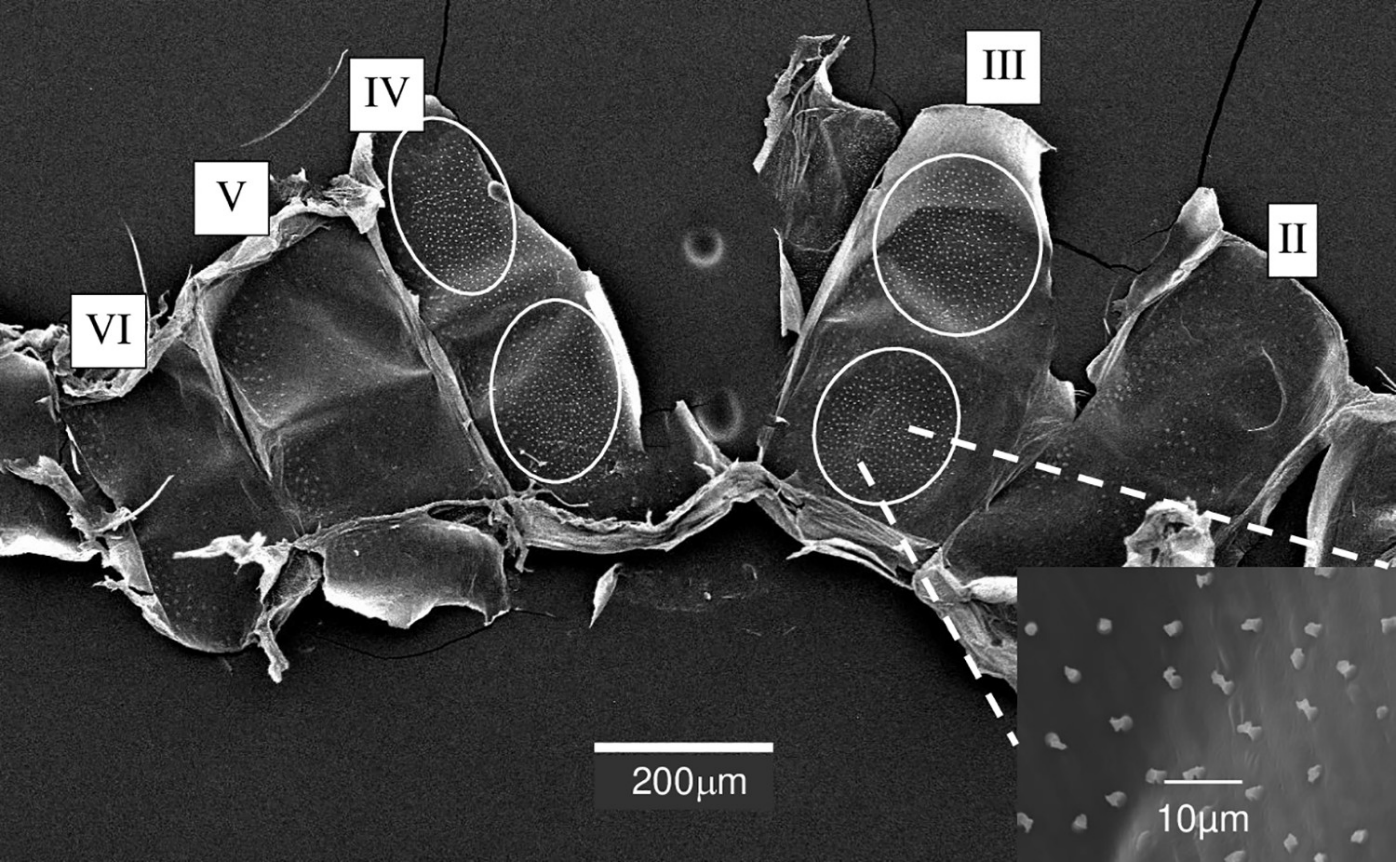
SEM of the interior cuticular surface of abdominal segments II-VI of *Lu*. *longipalpis* from Campo Grande showing the areas corresponding to pale patches normally seen from the exterior. Tergites II to VI are indicated by Roman numerals. The areas of the internal surface corresponding to the pale spots seen from the exterior area indicated by the white oval shapes. The insert is a close-up magnification of the cuticular structures associated with the secretory apparatus and seen within the oval-shaped (pale patch) areas.

Examination of the interior surface of the *Lu*. *longipalpis* abdomen showed structures that were distributed over an area that matched both the size and shape of the pale spots previously observed on the external surface of tergites III and IV [19,29,42] Fig 1. Density of these structures in the samples from Campo Grande was approximately 13/1000 μm^2^ (ca. 1627 structures in total), Jacobina 18/1000 μm^2^ (ca. 1415 structures in total) and Sobral 18/1000 μm^2^ (ca. 3469 structures in total).

**Fig 2:**
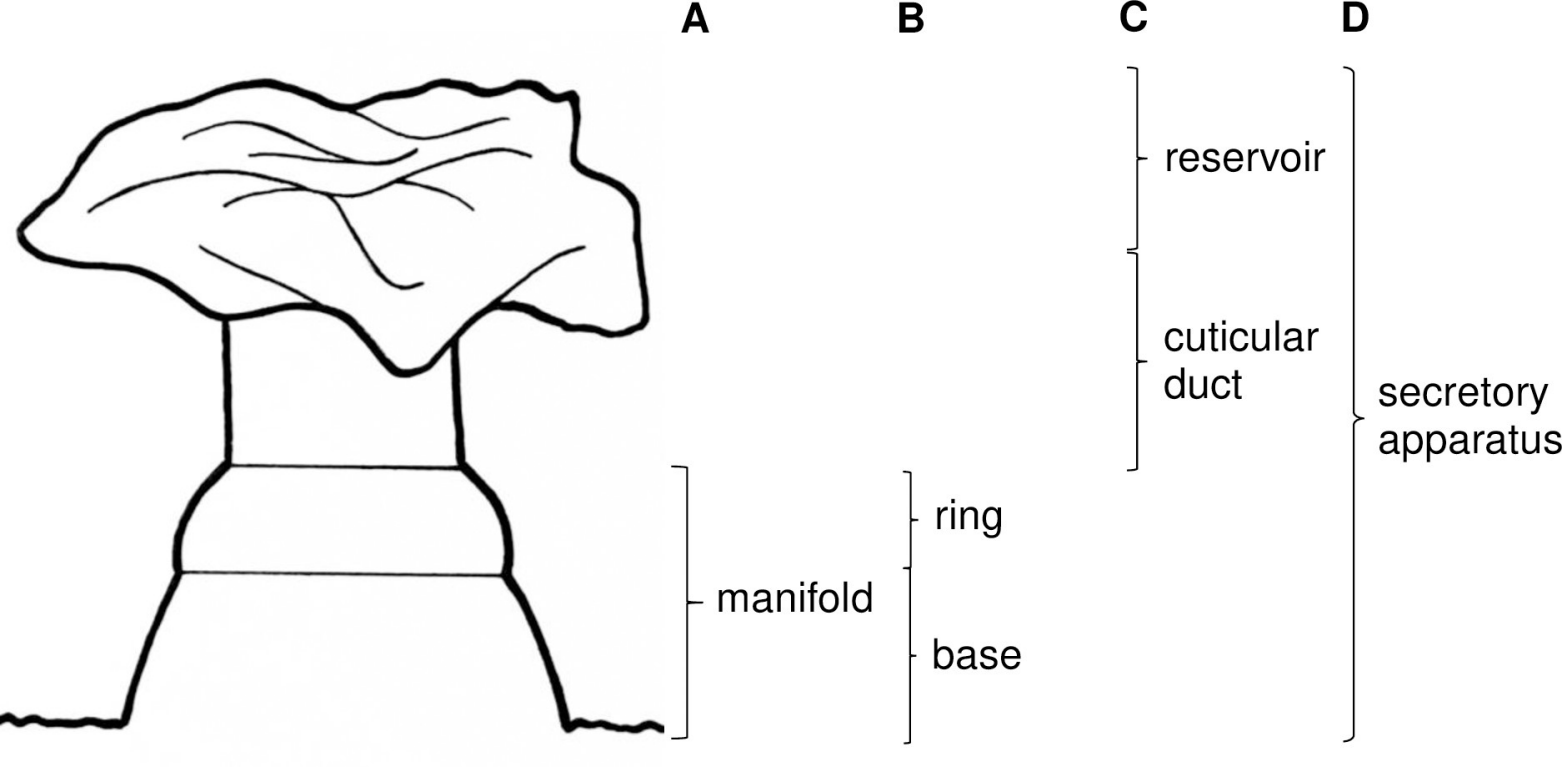
Drawing of the components of the secretory apparatus of *Lu*. *longipalpis* from Campo Grande, Brazil. **A)** manifold connected to the inner surface of the abdominal cuticle; **B)** components of the manifold, ring + base; **C)** secretory reservoir + cuticular duct; **D)** secretory apparatus; reservoir + cuticular duct + manifold.

Observation of the morphology of the internal cuticular structures which remained after KOH digestion indicated that two sections were present; the first was a section which connected to the interior wall of the tergite (or which is an extension of the tergite) and which we have called the manifold (Fig 2A). The manifold has two distinct parts; the base and a distally positioned section, the ring, which has the appearance of a doughnut shaped ring of thicker cuticle (Fig 2B). The second part of the whole structure is the cuticular duct (named the “chitinous duct” previously by [42], connected to the manifold at the proximal end and which terminates in the reservoir (this is surrounded by the cellular end apparatus) at the distal end (Fig 2C). The reservoir is seen to be a cuticular bag that can assume different shapes. Both the cuticular duct and the reservoir are structures that have been previously observed in TEM studies [27,29] but which have not been observed by SEM studies. All parts together can be described as the secretory apparatus (Fig 2D). In some cases, during the preparation of the samples the ductule/reservoir complex become detached from the manifold structure showing that the interior of the manifold appears to be hollow.

**Fig 3:**
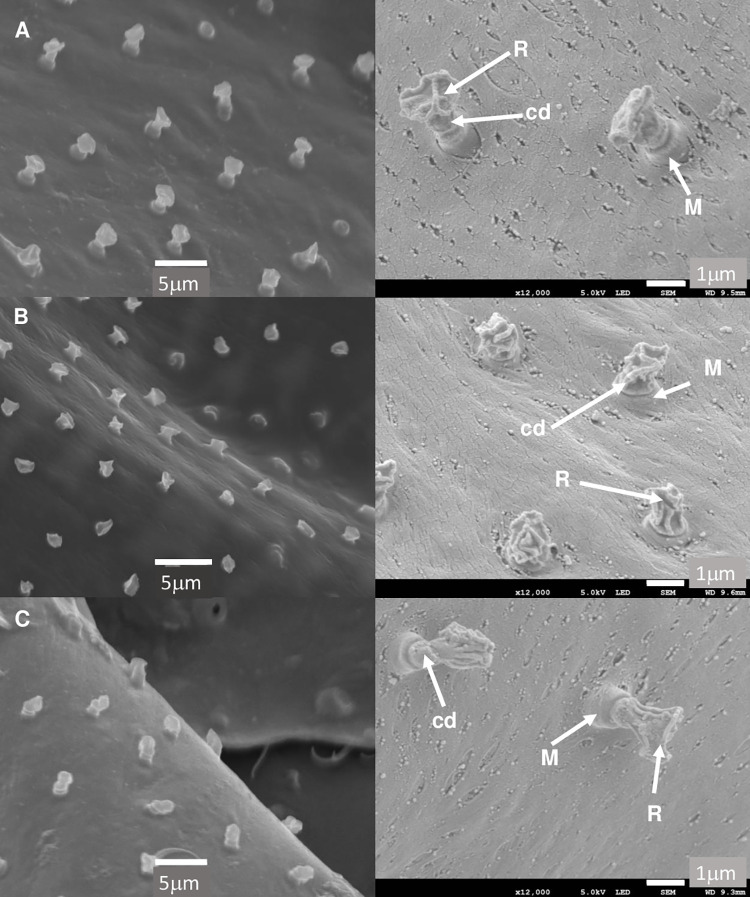
SEM images of the inner cuticle surface of the abdominal tergites of 3 members of the *Lu*. *longipalpis* s.l. species complex showing the cuticular elements; manifold, reservoir and cuticular duct, of the secretory apparatus. Secretory apparatus observed by SEM after KOH digestion of *Lu*. *longipalpis* abdominal tergites from; A) Campo Grande, B) Sobral and C) Jacobina. The Manifold (M), Reservoir (R) and cuticular duct (cd) are indicated in the enlarged images on the right of the figure. Images on the left side (x3,500 magnification) were taken on a Jeol JSM-5600. Images on the right (x12,000 magnification) were taken on a Jeol JSM-7800F.

The secretory apparatus of the three members of the *Lu*. *longipalpis* complex examined in this study are shown in Fig 3.

**Fig 4:**
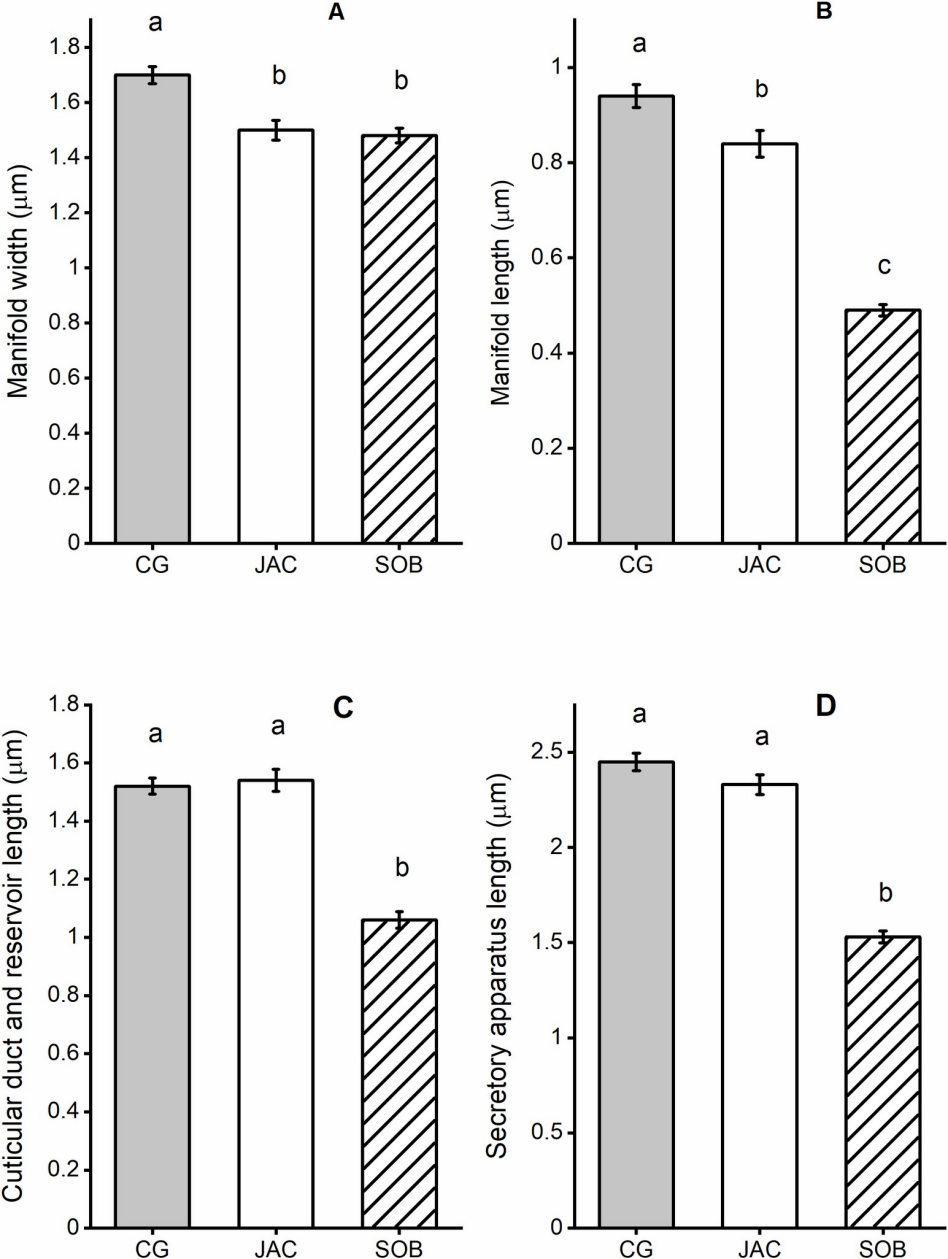
Dimensions of the components of the secretory apparatus observed in 3 members of the *Lu*. *longipalpis* s.l. species complex. Mean size of the measured structures (μm); manifold width (A), manifold length (B), reservoir and cuticular duct length (C) and secretory apparatus length (D) for each of the three members of the *Lutzomyia longipalpis* species complex; Campo Grande (CG), Jacobina (JAC) and Sobral (SOB). Error bars are ± standard error of the mean. Tukey’s test was used to compare sizes of structures between each member of the complex, measurements with the same letter (a, b or c) were not significantly different (P<0.05) from each other.

There was a highly significant difference in the widths of the manifolds (Fig 2) of the 3 members of the *Lu*. *longipalpis* complex (df = 147; F = 15.17; *P*<0.001). The Campo Grande manifold was significantly wider (mean±sem; 1.70±0.031μm) than either the Jacobina (1.50±0.036μm) or Sobral (1.48±0.027μm) manifolds which were not significantly different from each other (Fig 4A).

There was also a highly significant difference in the lengths of the manifolds (Fig 2) of the 3 members of the *Lu*. *longipalpis* complex (df = 147; F = 116.01; *P*< 0.001). The Campo Grande manifolds (0.94±0.024μm) were significantly longer than the Jacobina manifolds (0.84±0.028μm) which were significantly longer than the Sobral manifolds (0.49±0.012μm) (Fig 4B). There was also a significant difference in the lengths of the cuticular duct + reservoir (Fig 2) in the 3 members of *Lu*. *longipalpis* complex (df = 147; F = 75.55; *P* = 0.001). The Campo Grande and Jacobina cuticular ducts + reservoir were not significantly different from each other (1.52±0.027μm and 1.53±0.038μm respectively) whereas the Sobral ducts + reservoir were significantly shorter than the others (1.06±0.028μm) (Fig 4C). The overall length of the secretory apparatus (Fig 2) was also significantly different in the 3 members of the *Lu*. *longipalpis* complex (df = 147; F = 133.53; *P*<0.001). The Campo Grande secretory apparatus was similar in length to the Jacobina secretory apparatus (2.45±0.046μm and 2.33±0.051μm respectively). However, the Sobral secretory apparatus was significantly shorter than either Campo Grande or Jacobina (1.53±0.032μm) (Fig 4D).

**Fig 5:**
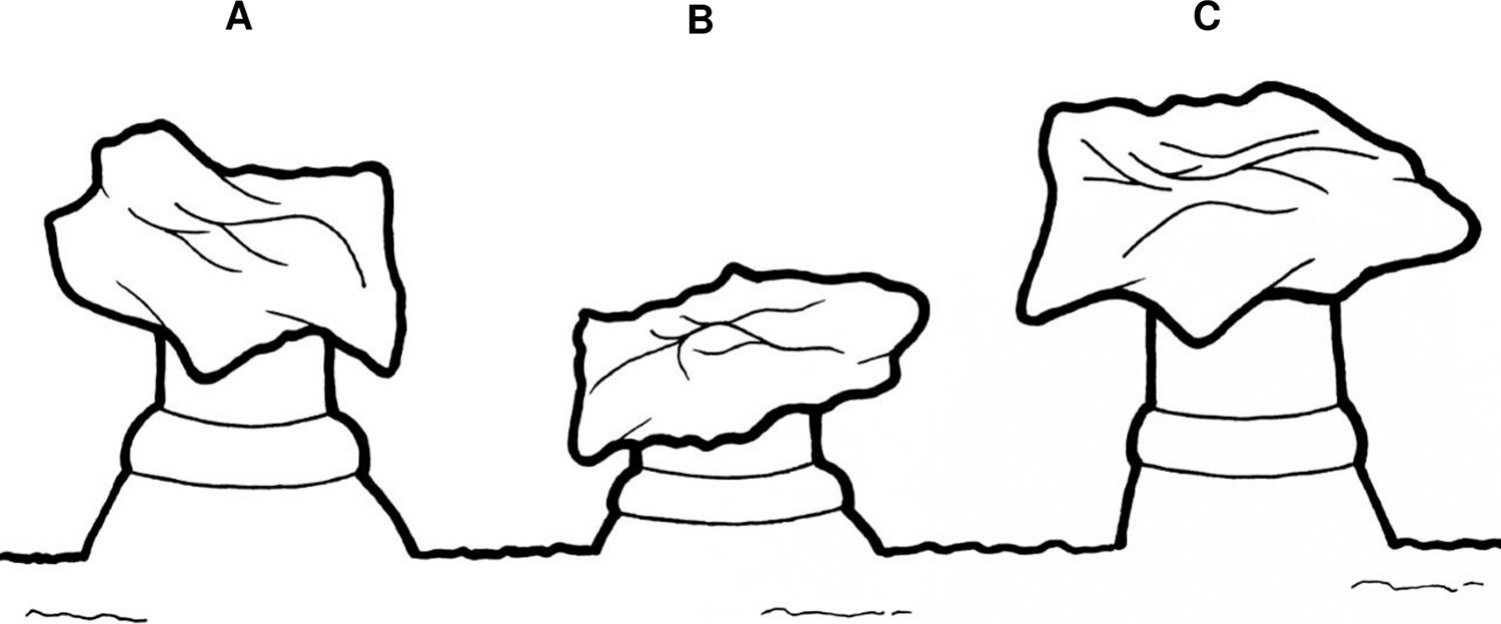
Drawing illustrating the morphological differences observed in the size and shape of the manifold in the three members of the *Lu*. *longipalpis* s.l. species complex. Campo Grande **(A)**, Sobral **(B)** and Jacobina **(C)**.

The differences in the size and shape of the secretory apparatus are summarised in Fig 5. The manifold of the Campo Grande (Fig 5A) member of the complex was longer and wider than the Jacobina type (Fig 5C). Overall, the secretory apparatus of the Sobral (Fig 5B) type was smaller than the others.

### *Migonemyia migonei* secretory apparatus

**Fig 6:**
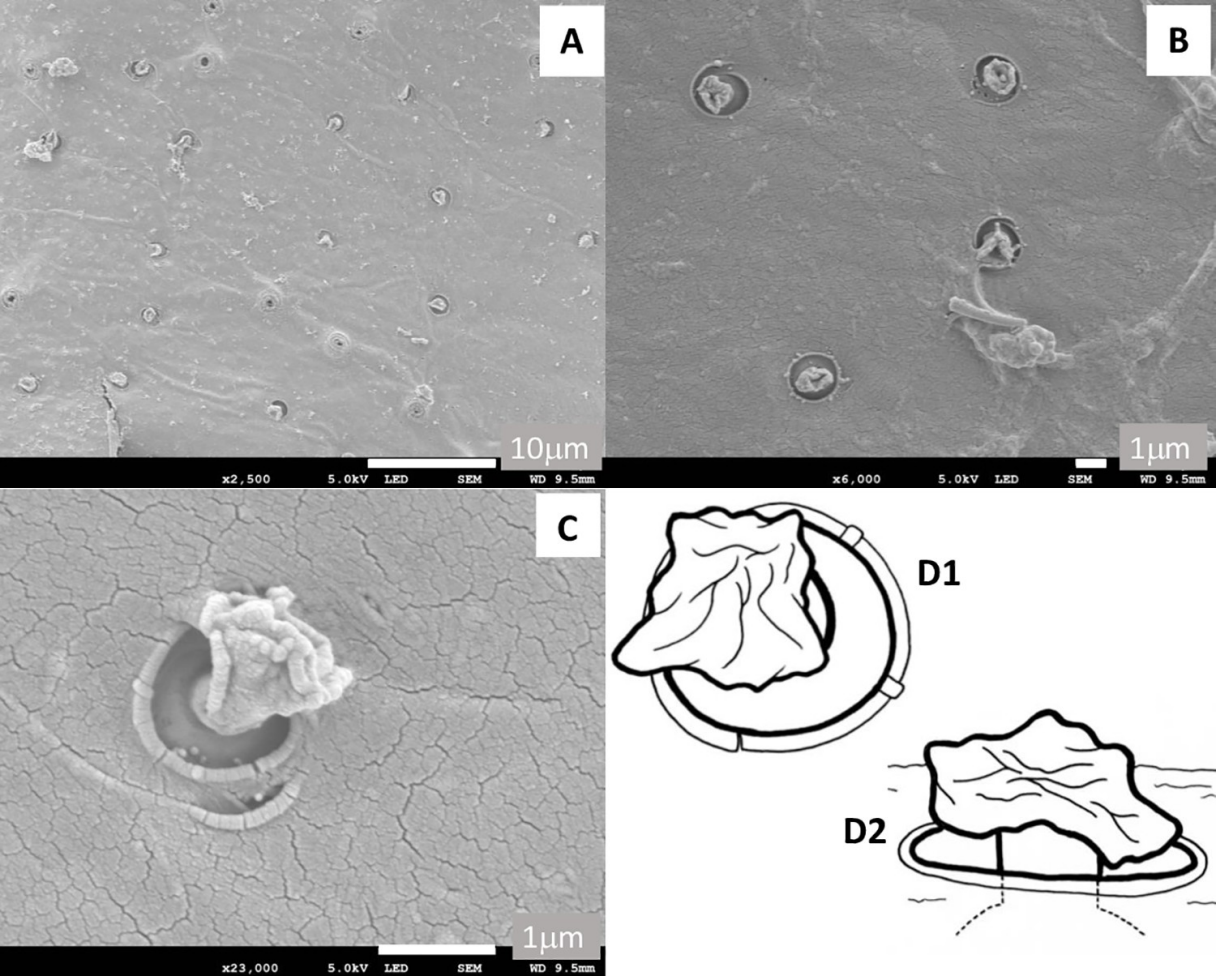
SEM of the interior surface *M*. *migonei* showing the observable cuticular elements of the secretory apparatus. **A)** Distribution of the secretory structures on the inner surface of the abdominal cuticle on tergite III, **B)** secretory apparatus set within a deep pocket embedded in the abdominal cuticle on tergite IV, **C)** close-up of a secretory unit showing the manifold embedded within the abdominal cuticle observed at the bottom of the pocket on tergite III. **D1)** Drawing of the *M*. *migonei* secretory apparatus from above (top left) showing the reservoir positioned over the hole in the abdominal cuticle and then **D2)** a side-on view showing the reservoir connected via cuticular to the top of the manifold sitting within the hole in the cuticle.

We found structures resembling the manifold, secretory duct and reservoir (ca. 9/1000μm^2^) previously seen in the *Lu*. *longipalpis* also present in the *M*. *migonei* samples. These structures were present on the internal cuticle surface of tergites III–VII. This distribution partially matched the distribution of the craters (with central pore and spike) previously reported on the external surface of tergites III-VI [37] (Fig 6A). The manifold was inserted within a deep recess (av. max width 1.50±0.04μm) and appeared to be embedded (ca. 0.25μm) within the cuticle. Only the reservoir appeared to be positioned fully within the interior of the abdomen (Figs 6B and 6C). Multiple observations of the manifolds from different positions suggest that it has the appearance illustrated in Fig 6D1 and 6D2.

## Discussion

Pheromone disseminating structures have been observed on the cuticle of 53 species of New World (*Lutzomyia* and *Brumptomyia* spp) and 5 Old World (*Sergentomyia*) species [13,19]. These structures take a diverse range of morphological forms and include structures such as pores in craters, pores with emergent spines, mammiform papules with or without spines and apple shaped structures [19]. This study reveals that in addition to the pheromone disseminating structures visible on the external surface of the abdomen and the elements (reservoir and cuticular duct) of the secretory apparatus observed by previous TEM studies [27,29,43] there are additional cuticular structures on the inside surface of the abdominal cuticle which have not been observed or described before. Each external structure is associated with a new internal structure which we have called the manifold (an engineering term to describe a device used in fluid or gas mechanics to aggregate or distribute gases or fluids). Although the precise function of the manifold is unknown it is connected via a tubule (cuticular duct) to the reservoir which is surrounded by the cellular secretory end-apparatus [27–29,43]. The manifold is thus clearly associated with the distribution of the pheromone from the secretory end-apparatus via the reservoir and cuticular duct to the external surface of the sand fly.

Scanning electron microscopy has been widely used to examine the external secretory apparatus and other externally visible cuticular structures in sand flies and other insects. The cells associated with pheromone production have been examined by TEM in sand flies [19,29,37,42,43] and other pheromone producing insect groups e.g. Lepidoptera, Coleoptera, Hymenoptera and species of Trichoptera from the families Rhyacophilidae and Limnephilidae [38,44–51]. Most of these studies were carried out to describe the arrangement, location and/or distribution of the pheromone gland secretory cells and were not carried out to examine the mechanisms by which the pheromone was transported from the site of biosynthesis to the point of dissemination on the surface of the cuticle. We are not aware of any published SEM studies that have examined the internal structures associated with pheromone production and transport in sand flies. The reservoir structures which can be seen in sand flies may also be seen in Blattaria: Blaberidae [52] and Coleoptera: Staphylinidae [53]. However, from the limited studies that have been undertaken in other insect species the manifolds appear to be unique to sand flies.

The *M*. *migonei* samples from the Charles University colony that had been stored in 70% ethanol required longer KOH digestion to remove the interior abdominal tissue than the freshly prepared *Lu*. *longipalpis* samples. Digestion for up to four hours was useful for *Lu*. *longipalpis* specimens and up to 10 hours was required for *M*. *migonei* specimens. For future studies freshly prepared samples are more likely to give better results than samples preserved in 70% ethanol.

In addition to the differences in the structure of their sex-aggregation pheromone, the members of the *Lu*. *longipalpis* s.l. species complex analysed in this study, have also shown differences related to the biosynthesis and release of their pheromones [54]. The results of this study also show significant morphological differences between the size and shape of the manifolds and the secretory apparatus overall. Interestingly there have been no reported differences in the size and shape of the papules which can be observed on the surface of the tergites in *Lu*. *longipalpis* s.l. The manifolds and other elements of the secretory apparatus of the Sobral member of the complex are significantly shorter than either Campo Grande or Jacobina. The manifold width of Sobral *Lu*. *longipalpis* is not significantly different to that of Jacobina but both are significantly narrower than in Campo Grande. The overall effect of the differences is that the Jacobina and Campo Grande are similar in size and shape to each other whereas the Sobral structure appears shorter and squatter. The effect of these differences may be to position the secretory cells that would surround the end apparatus closer to the surface in the Sobral member of the complex than the other two members. This may reflect the difference in the molecular weight and consequent volatility of the 2 methylsesquiterpenes (m.w. 218) found in Campo Grande and Jacobina compared to the increased molecular weight and decreased volatility of the diterpene (sobralene) pheromone (m.w. 272) found in Sobral. Thus, the distance for the larger molecule to travel from the secretory cell to the external surface is less than for the other two smaller and more volatile molecules. Alternatively, the differences may be related to phylogenetic differences in gland development [55] in different members of the *Lu*. *longipalis* species complex. Both hypotheses require further study to understand the situation in the Phlebotominae.

The manifold of *M*. *migonei* is very different to those observed in *Lu*. *longipalpis* s.l. and is positioned within the tergal cuticle in a pit-like structure. The reservoir is connected by a short duct to the manifold. The effect of this arrangement is that the secretory cells would be much closer to the surface than in *Lu*. *longipalpis* and this may reflect a relatively lower volatility (either higher molecular weight or presence of functional groups) of any sex aggregation pheromone produced by *M*. *migonei*. Although there is behavioural evidence for the presence of a sex-aggregation pheromone in *M*. *migonei*, no compound(s) with a chemical profile similar to the sex aggregation pheromones found in the *Lu*. *longipalpis* s.l. species complex has been found [37].

The density of manifolds found on the internal cuticle of sobralene producing Sobral (CE) *Lu*. *longipalpis* was 18 per 1000 μm^2^ (ca. 3469 in total) and matched the density of papules previously observed on the tergal surface of *Lu*. *longipalpis* from Sobral, (19 per 1000 μm^2^) [29]. This is not dissimilar to estimates of 14 per 1000 μm^2^ for the same Sobral population [28]. The density of manifolds in the Campo Grande (MS) (*S*)-9-methylgermacrene-B producing population was approximately 13 per 1000 μm^2^, part-way between the 8 per 1000 μm^2^ papules observed by Lane and Ward (1984) in *Lu*. *longipalpis* collected at Lapinha (MG) and 21 per 1000 μm^2^ papules in *Lu*. *longipalpis* also collected at Lapinha [29]. The meaning of this difference is unclear, it may be related to significant differences between the Campo Grande population and the Lapinha population, similar to those observed between the Sobral (*S*)-9-methylgermacrene-B and the Lapinha population in which the Sobral population was found to produce significantly more pheromone than the Lapinha population [24].

This is the first time that the manifold structure has been seen in any group of insects and its function is unclear. It may be that the manifold is only found in Phlebotomine sand flies, but it may occur in other insect orders. It could simply be a device to ensure the safe transport of the sex aggregation pheromone from the secretory cells through the cuticle. The sturdiness of the structure could suggest that it is designed to minimise possible leakage of potentially toxic terpene [56] pheromone into the abdomen. Male sand flies engage in combat with other males to defend territory and in these aggressive battles [57,58] males could potentially risk dislodging unprotected plumbing carrying pheromone without the additional support provided by the manifold. It would be worth examining the internal cuticular secretory structures of other insect groups where males produce sex/aggregation pheromones and fight with other males to defend territories e.g. *Frankliniella occidentalis* [59,60]. Without a clear view of the interior, it is uncertain if additional functionality may exist within the manifold e.g. a passive or controllable valve or a reservoir of pheromone or other mechanism to regulate pheromone flow to help provide a supply of pheromone when it is required [54]. In the future it may be possible to get a clear view of the interior of these structures using Synchrotron Radiation Microtomography [61].

Observing the location, distribution and density of the manifolds on the inner cuticle was a convenient way to check the whole inner cuticle of the abdomen for secretory structures. More studies should now be conducted to compare the number of these structures in different members of the *Lu*. *longipalpis* s.l. complex and from different parts of Brazil as well as to determine their distribution in other New and Old-World species.

The *M*. *migonei* manifold lay within the cuticle and although it was possible to observe it within the clearly defined recess in which it was positioned we could not check morphological details. The details of how the secretory apparatus is connected to the exterior remains elusive and although there was one manifold per external structure (“spined crater”) it was not possible to clarify if there was more than one opening per pheromone secreting structure through the cuticle [19] to the exterior of the insect. We found that the manifolds were distributed on tergites III to VII but more studies should be carried to fully describe the morphology of the manifold and then link the morphological form to the pheromone and its function.

These results may contribute to the discussion of the nature of the *Lu*. *longipalpis* species complex, as they show that there are clear morphological differences between 3 of the members of the complex. These structures may also be useful taxonomic tools more generally within the Phlebotominae. This study also shows that in addition to the widespread distribution of the external structures linked to pheromone release these internal structures are likely to be strongly associated with active pheromone production and release. Their presence in species where pheromone production has been inferred through behavioural studies but not confirmed through chemical analysis should be determined. Behavioural analysis for example, in the lab and field has shown that female *Phlebomomus papatasi* and *P*. *argentipes* are attracted to conspecific males [62–64], however no external structure, associated with pheromone release, has been observed on the abdomen. In addition, several species of sand flies have been found to have external structures that could be associated with pheromone release however no putative pheromone chemicals have been found [35]. The presence of the manifold and associated cuticular duct and reservoir is considerably easier to locate than hidden isolated external structures, as in *L*. *renei* [29], therefore locating the secretory apparatus and thus identifying which sand fly species may produce pheromone will be easier and may simplify the search for the pheromone source.
